# High-resolution analyses of the secretomes from murine C2C12 cells and primary human skeletal muscle cells reveal distinct differences in contraction-regulated myokine secretion

**DOI:** 10.3389/fphys.2025.1549316

**Published:** 2025-03-25

**Authors:** Pia Marlene Förster, Julian Hogenkamp, Moira Fee Pottgießer, Christian Binsch, Awovi Didi Humpert, Carolin Laura Brügge, Michelle Isabel Deatc, Regina Ensenauer, Alexandra Chadt, G. Hege Thoresen, D. Margriet Ouwens, Sonja Hartwig, Stefan Lehr, Hadi Al-Hasani

**Affiliations:** ^1^ Institute for Clinical Biochemistry and Pathobiochemistry, German Diabetes Center (DDZ), Leibniz Center for Diabetes Research at Heinrich Heine University, Medical Faculty, Düsseldorf, Germany; ^2^ German Center for Diabetes Research (DZD e.V.), Düsseldorf, Germany; ^3^ Institute of Child Nutrition, Max Rubner-Institut, Federal Research Institute of Nutrition and Food, Karlsruhe, Germany; ^4^ Section for Pharmacology and Pharmaceutical Biosciences, Department of Pharmacy, University of Oslo, Oslo, Norway; ^5^ Department of Pharmacology, Institute of Clinical Medicine, University of Oslo, Oslo, Norway; ^6^ Department of Endocrinology, Ghent University Hospital, Ghent, Belgium

**Keywords:** HSkMCs, C2C12, myokines, EPS, muscle contraction, mass spectrometry, secretomes

## Abstract

Myokines released by skeletal muscle in response to contraction may contribute to the health-promoting effects of exercise. Previous studies with cultured rodent and human myotubes have revealed highly complex patterns of myokine secretion. However, the commonalities and differences in the secretory response of the different cell models have not been explored, limiting the interpretation of these results. In the present study, we performed a comprehensive analysis of contraction-regulated secretomes using the most commonly used skeletal muscle cell models, cultured murine C2C12 myotubes and satellite cell-derived primary human myotubes (HSkMC). The cells were subjected to low-frequency electrical pulse stimulation (EPS) for 6 h followed by high-resolution mass spectrometry analysis of secreted proteins in the culture medium. We identified 5,710 and 3,285 proteins in the secretomes of C2C12 myotubes and HSkMC, with 80% of human myokines also detected in the murine secretome. Additionally, we found 518 and 336 secreted proteins that were differentially regulated during contraction in murine and human cells, respectively, along with 1,440 and 385 previously unknown potential myokines secreted by murine and human myotubes. Bioinformatic prediction analyses revealed that the majority of the newly identified myokines were secreted via unconventional protein secretion pathways (UPS) in the murine secretome, whereas most novel proteins in the human secretome were secreted via the classical endoplasmic reticulum (ER)-to-Golgi pathway. Moreover, ontology analysis indicates cell type-specific differences in cellular compartments involved in myokine secretion. Collectively, our results provide a comprehensive overview of the secretomes of two of the most commonly used cell models and may provide guidance for further studies of myokines.

## Introduction

Skeletal muscle is a very dynamic and plastic tissue that accounts for about 40% of total body mass in humans. It is a versatile organ involved in many immunometabolic processes, such as the storage of amino acids and carbohydrates. Additionally, it is part of the locomotor system responsible for the movement of the joints by contraction (Florin et al., 2020). It contains 50%–75% of all body proteins and functions as a highly metabolically active organ, secreting muscle-specific proteins that exert auto-, para- and endocrine effects (Frontera and Ochala, 2015). Pedersen et al. were the first who proposed to define these proteins as myokines, which act as mediators and enable the skeletal muscle to participate in the inter-organ crosstalk by communicating with other cells (Pedersen et al., 2007). Myokines are involved in multiple endogenous processes such as metabolic regulation of lipid and glucose metabolism, inflammatory processes, angiogenesis and myogenesis (Laurens et al., 2020). In previous studies, the myokine profile has been investigated in detail by performing non-targeted mass spectrometry (MS)-based analyses, using immortalized, rodent C2C12 and L6 skeletal muscle cell lines or primary human skeletal muscle cells (HSkMCs) (Florin et al., 2020). Both C2C12 and L6 cells are immortalized cell lines, derived from skeletal muscle of mouse and rat, respectively, established by Yaffe (1968), Yaffe and Saxel (1977). Importantly, the myoblast precursor cells can be differentiated in culture into multinucleated, contractile myotubes that exhibit many biochemical, morphological, and metabolic features of adult skeletal muscle (Aas et al., 2013). Investigation of myokine secretion profiles during and after myogenesis has identified hundreds of secreted proteins from C2C12 cells and HSkMCs that may have functions in signal transduction, extracellular matrix (ECM) formation and remodeling, muscle development and neurogenesis (Henningsen et al., 2010; Le Bihan et al., 2012; Hartwig et al., 2014; Grube et al., 2018). Recently, two studies have analyzed the contraction-induced secretome of murine C2C12 cells (Gonzalez-Franquesa et al., 2021) and primary HSkMCs (Mengeste et al., 2022) that were subjected to different protocols of chronic low-frequency electrical pulse stimulation (EPS), a widely used *in vitro* method to induce muscle contraction. Both studies identified a large number of proteins in the conditioned cell culture media with 75 (C2C12) to 149 proteins (HSkMCs) that were differentially regulated in response to chronic low-frequency EPS (>24 h) (Gonzalez-Franquesa et al., 2021; Mengeste et al., 2022). In fact, C2C12 cells and HSkMCs are among the most commonly used cell models to study myokine secretion and to analyze contraction-induced changes in the muscle secretome. However, relatively little is known about the extent to which the secretomes of C2C12 cells and primary HSkMCs are similar or distinct. Adding to the complexity, very different EPS protocols have been used in the previous studies (Nikolić et al., 2017), making it more difficult to compare C2C12 cells and primary HSkMCs in their secretory response to contraction (Gonzalez-Franquesa et al., 2021; Mengeste et al., 2022). Accordingly, Abdelmoez et al. have observed striking differences between these cell models by comparing the transcriptomes of C2C12, L6, and primary human myotubes after exposure to an acute low-frequency (3 h) EPS protocol (Abdelmoez et al., 2020). The reported differences at the transcriptome level between these 2 cell models after exposure to EPS suggest that the EPS-induced secretomes of C2C12 cells and HSkMCs may also differ substantially from each other. Therefore, we aimed to comprehensively analyze and compare the muscle secretomes of the most commonly used cell models. We subjected differentiated murine and human myotubes to acute low-frequency EPS (6 h) and subsequently analyzed the conditioned media (CM) by high-resolution MS.

## Materials and methods

### Cell culture: murine skeletal muscle cells

C2C12 myoblasts were proliferated in Dulbecco´s Modified Eagle Medium (DMEM; Gibco, ThermoFisher Scientific, Cat. No.: 11995065) containing 4.5 g/L (25 mM) D-Glucose, 10% fetal bovine serum (FBS) (Sigma-Aldrich, Cat. No.: F7524), 4 mM L-glutamine, 1 mM sodium pyruvate and 1% penicillin-streptomycin (100 U/mL, 100 μg/mL; Gibco, ThermoFisher Scientific, Cat. No.: 15140122) at 37°C in a humidified atmosphere (80%–90%) with 5% CO_2_ in air. Growth medium was changed every 3 days. At approximately 70% confluency, cells were seeded into 6-well plates (Greiner, Cat. No.: M8562) at a density of 200,000 cells per well. Following standard protocols, differentiation to myotubes was initiated 1 day after seeding by reducing the amount of serum in the medium to 2% horse serum (ATCC, Cat. No.: 302040) as described (Benninghoff et al., 2020). Viability of the cells was assessed by trypan blue staining (Gibco, ThermoFisher Scientific, Cat. No.: 15250–061), morphological inspection and lactate dehydrogenase (LDH) release assay of culture media (LDH-Glo™ Cytotoxicity Assay, Promega, Cat. No.: J2380), according to the manufacturer instructions. Medium was changed after 3 days and all experiments were performed on the sixth day of differentiation as described (Benninghoff et al., 2020).

### Cell culture: primary human skeletal muscle cells

Primary human skeletal muscle myoblasts from *quadriceps femoris* muscle of healthy male subjects (Lonza, Cat. No.: CC-2580; Age: 16–35 years, BMI: 19–26 kg/m2) were cultivated in skeletal muscle myoblast growth medium (SkGM™-2, Lonza, Cat. No.: CC-3245) at 37°C in a humidified atmosphere (80%–90%) with 5% CO_2_ in air. Growth medium was changed every 2 days until 60%–65% confluency was reached. Cells were seeded in 6-well plates at a density of 200,000 cells per well. At approximately 90%–95% confluency, differentiation to myotubes was initiated by switching to DMEM supplemented with 1 g/L (5.55 mM) D-glucose (Gibco, ThermoFisher Scientific, Cat. No.: 11054001), 1 mM sodium pyruvate, 1% penicillin-streptomycin (100 U/mL, 100 μg/mL; Gibco, ThermoFisher Scientific, Cat. No.: 15140122), 2% FBS (Sigma-Aldrich, Cat. No.: F7524) and 2% GlutaMAX™ (Gibco, ThermoFisher Scientific, Cat. No.: 35050038). Viability of the cells was assessed by trypan blue staining, morphological inspection and LDH release assay of culture media (LDH-Glo™ Cytotoxicity Assay, Promega, Cat. No.: J2380), according to the manufacturer instructions. Medium was changed every 2 days and all experiments were performed on day six of differentiation as described (Hartwig et al., 2014).

### Electrical pulse stimulation

On day six of the differentiation, myotubes (C2C12 and HSkMCs) were washed three times with Dulbecco’s phosphate-buffered saline (DPBS, Gibco, ThermoFisher Scientific, Cat. No.: 14190144) and medium was changed to serum- and phenol red-free DMEM (Gibco, ThermoFisher Scientific, Cat. No.: 11054020 containing 1 g/L (5.55 mM) (HSkMCs) or 4.5 g/L (25 mM) (C2C12) D-Glucose enriched with 1 mM sodium pyruvate, 1% penicillin-streptomycin (100 U/mL, 100 μg/mL; Gibco, ThermoFisher Scientific, Cat. No.: 15140122), and 4 mM GlutaMAX™ (Gibco, ThermoFisher Scientific, Cat. No.: 35050038). Carbon electrodes (C-Dish™, IonOptix LLC) were pre-soaked in a serum-free medium, then placed on a culture dish touching the medium and put into an incubator. Myotubes were stimulated in serum-free medium with EPS (C-Pace EP, IonOptix LLC) for 6 hours with 2 m pulses at 40 V (HSkMCs) and 11.5 V (C2C12) following the most frequently used established protocols for these cell types (Nikolić et al., 2017). Control cells, also exposed to electrodes but not connected to the power supply, were simultaneously serum-starved for 6 hours. Eventually, CM of stimulated and unstimulated cells was collected and centrifuged at 1,000 x g for 10 min at 4°C to remove debris and apoptotic cells. Cell integrity was unaffected by EPS as assessed through measuring LDH in the CM of sedentary and contracted cells (data not shown). Eventually, the cells were washed once with cold DPBS and lyzed either in denaturing SDS (sodium dodecyl sulfate) buffer (100 mM Tris-HCl, 4% SDS and 100 mM DTT, supplemented with cOmplete™ (Roche, Cat. No.: 04693132001) for proteome analysis or in Western blot lysis buffer (20 mM Tris-HCl, 150 mM NaCl, 1 mM EDTA, 1 mM EGTA, 1% Triton X-100, supplemented with cOmplete™ and PhosSTOP™ (Roche, Cat. No.: 04693132001, 4906845001) for immunoblot analysis. All samples were stored at −80°C until further analysis.

### Sample preparation for MS-based secretome analyses of C2C12 cells and HSkMCs

**FIGURE 1:**
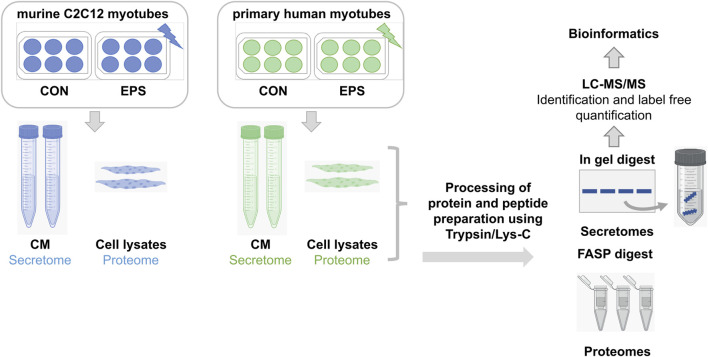
Bottom-up proteomics workflow of secretome and proteome analyses. Murine C2C12 and primary human myotubes were subjected to acute low-frequency EPS. CM from stimulated and unstimulated cells were collected for secretome analysis, whereas only unstimulated cells were lyzed and harvested for cellular proteome analysis. Proteins were digested using trypsin/Lys-C mix prior to high-resolution LC-MS analysis. Samples from C2C12 cells (*n* = 3) are demonstrated in blue, while samples from HSkMCs from three different donors (*n* = 3) are shown in green. CON: control, CM: conditioned media, EPS: electrical pulse stimulation, LC: liquid chromatography, MS: mass spectrometry.

For secretomic profiling of the CM of EPS-treated C2C12 cells and primary HSkMCs, protein load for C2C12 supernatants was adjusted to 25 μg, whereas for HSKMCs supernatant 4.5 µg protein were loaded onto a sodium dodecyl sulfate polyacrylamide (SDS-PAGE) gel (10% polyacrylamide, 0.5 cm separation distance) and subjected to in-gel protein digestion as previously described (Hartwig et al., 2019; Apostolopoulou et al., 2021) (Figure 1). Briefly, gel slices were alternated washed with 25 mM ammoniumbiocarbonat and 50% ACN (v/v). Proteins were reduced with dithiothreitol (DTT) (65 mM for 10 min at 95°C) and alkylated with 216 mM Iodacetamid for 15 min at RT in the dark. Follwed by an additional washing step, gel slices were shrinked with 100% ACN and digestion with 400 ng Trypsin/Lys-C (Promega, Cat. No.: V5071) was performed over night at 37°C. Peptides were eluted with 1% TFA (v/v) and 1% TFA/90% ACN (v/v). Peptides were lyophilised and stored at 4°C. For MS-based secretome analyses, lyophilized peptides were reconstituted in 1% trifluoroacetic acid (TFA) (v/v) and separated by liquid chromatography (LC) (UltiMate™ 3,000, ThermoFisher Scientific), connected to an Orbitrap Exploris™ 480 mass spectrometer (ThermoFisher Scientific). Peptides were trapped and desalted on an Acclaim™ PepMap™ C18-LC-column (ID: 75 μm, 2 cm length; ThermoFisher Scientific), followed by separation on an Aurora C18 column (AUR2-25075C18A, 25 cm × 75 μm C18 1.6 µm; IonOpticks). Monitoring performance of chromatographic separation was done by running HeLa samples (50 ng) supplemented with iRT-peptides (Biognosys) analyzed using QuiC-Software (Biognosys). For the separation, a 2 hours three-step gradient at a total flow rate of 300 nL/min with buffer A (0.1% formic acid) and buffer B (80% ACN, 0.1% formic acid) was used. First, linear from 2%–19% buffer B for 72 min, second from 19%–29% buffer B for 28 min, then from 29%–41% buffer B for 20 min and a 1 minute linear gradient increasing buffer B to 95%. The MS data were acquired in data-dependent acquisition (DDA) mode at 120,000 resolution (2 s cycle time), m/z range of 350–1,200 and a normalized automatic gain control (AGC) target value of 300%. Fragmentation precursor selection filters were set to charge state between two and six and dynamic exclusion of 45 s. Fragmentation of precursors was done with an isolation window (m/z) of 1.6, higher-energy collisional dissociation (HCD) energy of 30% at 15,000 resolution with automatic adjustment of AGC target value and injection time.

### Sample preparation for MS-based proteome analyses of C2C12 cells and HSkMCs

Cell lysates from unstimulated C2C12 cells and primary HSkMCs were further solubilized in denaturing SDS buffer (100 mM Tris-HCl, 4% SDS and 100 mM DTT, supplemented with Complete™ (Roche, Cat. No.: 04693132001), by 10 strokes through an insulin syringe (needle 26 gauge), followed by sonification (two times pulse 0.09 s_10 s) for proteome analyses (Figure 1). After centrifugation at 75,000 x g for 30 min at 4°C the supernatants were transferred to new reaction tubes. Protein concentrations were determined by direct photometric measurements (NanoDrop™ spectrophotometer, ThermoFisher Scientific). To adjust for SDS interference, we included blank controls of the lysis buffer without protein as the reference. Protease digestion was performed according to the filter-aided sample preparation (FASP) procedure (Wiśniewski et al., 2009) with some adaptations. Shortly, protein samples (100 µg) were incubated at 96°C for 10 min and then diluted 1:10 (v/v) with urea buffer (UB: 8 M urea, 100 mM Tris-HCl pH 8). Samples were then centrifuged at 10,000 x g using centrifugal filter devices (Amicon® Ultra-0.5, P/N UFC5030, Merck) and alkylated by incubation with 50 mM iodoacetamide for 15 min at room temperature. Washing steps with urea buffer were repeated three times (repeated centrifugation at 10,000 x g for 16 min), protein lysates were digested using Trypsin/Lys-C mixture (Promega, Cat. No.: V5071) in a 1:25 (w/w) enzyme/protein ratio overnight at 37°C. Peptides were collected by centrifugation through the filter device. The next day, filter devices were centrifuged and the peptides were collected and acidified with TFA to a final concentration of 0.1% (v/v). Purification was obtained using C18 solid phase extraction (Strata C18-E, 200 mg/1 mL, Phenomenex), according to the manufacturer’s instructions. Briefly, after activation with 300 µL methanol, columns are washed (300 µL 80% acetonitrile in 0.1% TFA) and equilibrated two times using 300 µL 0.1% TFA. Subsequently, sample and flowthrough is loaded one after the other. After washing the cartridge with 300 µL 0.1% TFA, peptides are eluted 2 times with 200 µL (60%, acetonitrile in 0.1% TFA). Eluates were lyophilized and stored as aliquots at −80°C. For LC-MS/MS analyses, lyophilized peptides were reconstituted in 1% TFA (v/v) and peptide concentrations were determined using Quantitative Colorimetric Peptide Assay (Pierce™, ThermoFisher Scientific, Cat. No.: 23275). Lyophilization was carried out at 45°C in a SpeedVac concentrator (ThermoFisher Scientific). Peptide concentrations were determined before MS analysis. Samples (400 ng) were run in triplicates by LC (UltiMate™ 3,000, ThermoFisher Scientific) as described above. MS analysis was performed on an Orbitrap Fusion™ Lumos™ mass spectrometer (ThermoFisher Scientific) coupled to a Nanospray Flex™ ion source and equipped with a high-field asymmetric waveform ion mobility spectrometry (FAIMS Pro) interface. MS-data were obtained in DDA mode using FAIMS compensation voltages (CV) of −40, −60 and −80 V. MS spectra were acquired at 120,000 resolution (3 s cycle time) and m/z range of 350–1,600. Fragmentation precursor selection filters were set to charge state between two and seven, dynamic exclusion of 30 s and intensity threshold of 2.5 × 10^4^. Fragmentation of precursors was done with an isolation window (m/z) 3.6, HCD energy of 30% at 15,000 resolution with automatic adjustment of AGC target value and injection time.

### In-silico MS data analysis

Proteome Discoverer™ (PD™) 3.0 software (ThermoFisher Scientific) was used to analyze the MS raw files. For spectral recalibration, the SpectrumRC node was used with the FASTA database (UniProtKB database, reviewed SwissProt, *Homo sapiens* TaxID = 9,606, v2022-12-14 and v2023-06-28, *Mus musculus* TaxID = 10090, v2023-03-01). The minora feature detector node was used for quantification with default settings (minimum trace length 5, max. delta RT of isotope pattern multiplets of 0.2 min and for feature to ID linking use only high confident peptide spectrum matches (PSMs). CHIMERYS (Burger, 2022) search was performed against UniProtKB database (reviewed SwissProt, *Homo sapiens* TaxID = 9,606, v2022-12-14 and v2023-06-28, *Mus musculus* TaxID = 10090, v2023-03-01 and an in-house contaminant fasta file). Enzymatic digest was conducted using trypsin, allowing a maximum of two missed cleavage sites, b and y ions were selected for HCD fragmentation with a fragment mass tolerance of 0.02 Da. Carbamidomethylation of cysteine was set as static modification, while N-terminal acetylation, N-terminal methionine loss, N-terminal methionine loss/acetylation and methionine oxidation were allowed as variable modifications. Percolator (included in PD, max delta Cn: 0.01) was applied for data validation. Label-free quantification was performed on precursor intensities present in at least 20% of replicate features. The mass spectrometry proteomics data are deposited to the ProteomeXchange Consortium via the PRIDE with the dataset identifier PXD058612 and PXD059003.

### Bioinformatic analyses of MS-based data

The MS data were filtered to include (1) “master proteins” (proteins with the longest sequence were selected as master, when multiple proteins with the same score, same number of PSMs and peptides matched); (2) proteins with false discovery rate (FDR) < 0.01 for peptide identification (i.e., high confidence), (3) proteins with at least “one unique peptide”; (4) “species map” for either “*Homo sapiens*” or “*Mus musculus*” in PD software. Potential myokines were then analyzed using bioinformatic prediction tools, including SignalP 6.0 (Teufel et al., 2022), SecretomeP 2.0 (Bendtsen et al., 2004) and Outcyte 1.0 (Zhao et al., 2019). The SignalP algorithm screens for proteins that carry a signal peptide sequence at the N-terminus and are therefore predicted to be secreted via the classical ER-to-Golgi pathway. SecretomeP (nn-score<0.6) and Outcyte predict putative secretory proteins that are secreted via unconventional secretory pathways, such as intracellular- or transmembrane proteins. Moreover, Gene Ontology cellular component (GOCC) analyses were performed using generic GO term mapper (Boyle et al., 2004). Comparative analysis between C2C12 cells and primary HSkMCs were performed on the basis of gene symbols, and comparative analyses within the same species were performed on the basis of primary UniProtKB accessions. For further analyses of the large MS data sets, Venn diagrams were created using the web-based tool InteractiVenn (Heberle et al., 2015).

### Cross-species comparisons and literature searches

The literature search was performed using the PubMed^®^ database. For the comparative analyses, only papers published in English that performed non-targeted secretome analyses of skeletal muscle cells (C2C12 or primary HSkMCs) were considered. Keywords such as “secretome analysis”, “secreted proteins”, “mass spectrometry”, “skeletal muscle cells”, “myokines”, “exercise” and “electrical pulse stimulation” were used for PubMed^®^ database search. The available data from previous secretome studies provided a diverse and broad data set. The comparison between C2C12 cells and primary HSkMCs was performed using gene symbols. To increase data availability and transparency of the method, we provided our own secretome data and the comparison with data from the literature (Supplemental Table S5).

### Immunoblotting and immunoassays

After exposure to acute low-frequency EPS (6 h), C2C12 and HSkMCs lysates were homogenized for 10 min at 4°C in Uniprep Gyrator-24 (UniEquip) and centrifuged at 25,000 x g for 10 min at 4°C. Clear supernatant containing proteins was transferred to a new reaction tube and used for the following assay. Protein lysates (10–20 µg) were separated by denaturating SDS-PAGE, using 12% horizontal gels. After gel electrophoresis, samples were transferred to a nitrocellulose membrane (Amersham, Cat. No.: 10600002; 0.2 A, 2 h, 4°C). Membrane was blocked with Tris-buffered saline containing 0.05% Tween-20 (TBS-T) and 5% powdered milk, and incubated overnight with diluted antibodies against AMPKα (Cell Signaling, #2532), pAMPKα-Thr172 (Cell Signaling, #2531) and GAPDH (Cell Signaling, #2118). Washing steps were conducted three times with T-BST between incubations with antibodies. Primary antibodies were detected by HRP-conjugated secondary anti-rabbit antibody and signals were visualized with ECL reagent (Perkin Elmer, Cat. No.: NEL121001EA) in the ChemiDoc XRS + transilluminator (BioRad). Quantitative analysis was conducted by using Image Lab™ 6.0.1 software. Measurements for IL-6 in conditioned media were conducted using the Milliplex MAP Human Myokine Magnetic Bead Panel (Merck, Cat. No.: HMYOMAG-56K) and Milliplex Mouse Myokine Magnetic Bead Panel (Merck, Cat. No.: MMYOMAG-74K) with a Bio-Plex 200 System.

### RNA extraction, cDNA synthesis and reverse transcription quantitative real-time PCR (RT-qPCR)

To validate differentiation from myoblasts to myotubes, murine C2C12 cells and primary HSkMCs were seeded into 6-well plates at a density of 200,000 cells/well. At 90%–95% confluence, differentiation was initiated by switching from growth medium to differentiation medium. For harvest of myoblasts (day zero of differentiation), cells were washed once with cold PBS, lyzed in RLT-buffer (Qiagen, Cat. No.:74106) with 120 µL/well, shock-frozen in liquid nitrogen and stored at −20°C until further analysis. Differentiation medium was changed every two to 3 days until harvest of myotubes (day six of differentiation), which was conducted in the same procedure as for the myoblasts. RNA extraction was performed using RNeasy-Mini Kit (Qiagen, Cat. No.:74106) and cDNA synthesis was accomplished using the GoScript™ Reverse Transcriptase System (Promega, Cat. No.: A5001) with hexanucleotide primers (Roche, Cat. No.: C1181), following the manufacturer’s instructions. SYBR®Green self-designed Primers (Supplemental Table S1) for the respective genes were used for determination of gene expression by quantitative real-time PCR (qRT-PCR). Gene expression was determined using the ∆∆Ct method and *TATA-Box binding protein (Tbp)* was used as a housekeeping gene for C2C12 cells and *beta-2-microglobulin* (*B2M)* for primary HSkMCs.

### Statistical analysis

P-values for protein abundance ratios were derived from paired t-test and adjusted p-values were calculated using the Benjamini–Hochberg method. For identification of peptides and proteins, we used a threshold for false discovery rate (FDR) ≤ 0.01 using the “Protein FDR Validator node” in PD. Data for immunoblotting and RT-qPCR experiments are shown as means ± SEM and were analyzed by unpaired t-test (Welch´s correction) as indicated in Figure legends. P-values ≤0.05 were considered as threshold for statistical significance.

## Results

### Characterization of murine C2C12 cells and primary human skeletal muscle cells

**FIGURE 2:**
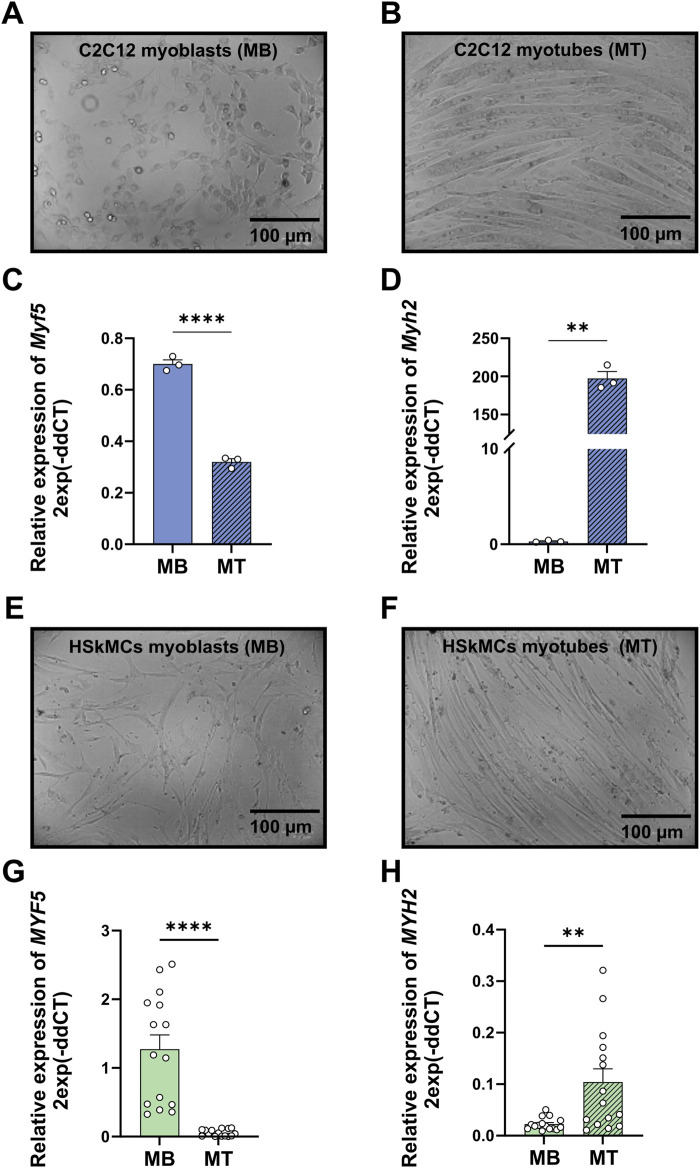
Validation of differentiation from murine C2C12 and primary human myoblasts to myotubes. Bright-field microscopy images of C2C12 myoblasts **(A)** and myotubes **(B)** RT-qPCR was performed to measure gene expression profiles of representative genes for myoblasts (*Myf5*) at day zero of differentiation and myotubes (*Myh2*) at day six of differentiation. mRNA levels of *Myf5* **(C)** and *Myh2* **(D)** measured in C2C12 muscle cells. Bright-field microscopy images of human myoblasts **(E)** and myotubes **(F)**. mRNA levels of *MYF5* **(G)** and *MYH2* **(H)** measured in primary HSkMCs. Quantified data was normalized to housekeeping genes *B2M* (HSkMCs) and *Tbp* (C2C12). Data are means ± SEM from three different individuals (*n* = 3) and C2C12 cells from three different experiments (*n* = 3) and were analyzed by unpaired *t*-test (Welch´s correction), *p < 0.05, **p < 0.01, ****p < 0.0001. (scale bars = 100 µM). Blue bars represent C2C12 myoblasts and green bars represent human myoblasts, while blue stripedbars display C2C12 myotubes and green stripedbars human myotubes. MB: myoblasts; MT: myotubes, SEM: standard error of the mean.

To monitor myogenic differentiation in both cell models, gene expression levels of *myogenic factor 5* (*Myf5*) as a marker for the onset of myogenesis and *myosin heavy chain 2* (*Myh2*) as a marker for later stages of differentiation were studied using RT-qPCR. Murine C2C12 and primary human myoblasts were harvested at day zero of differentiation. Both cell types showed significantly higher expression levels of *Myf5* in myoblasts compared to myotubes (Figures 2C, G). After 6 days of differentiation, both cell models showed a significant increase in gene expression of *Myh2* in myotubes compared to myoblasts (Figures 2D, H). Moreover, bright-field microscopy revealed similar differentiation of mononucleated myoblasts (Figures 2A, E) into fused, multinucleated myotubes (Figures 2B, F) after 6 days of differentiation for both cell models.

### Characterization of murine and human muscle secretomes

**FIGURE 3:**
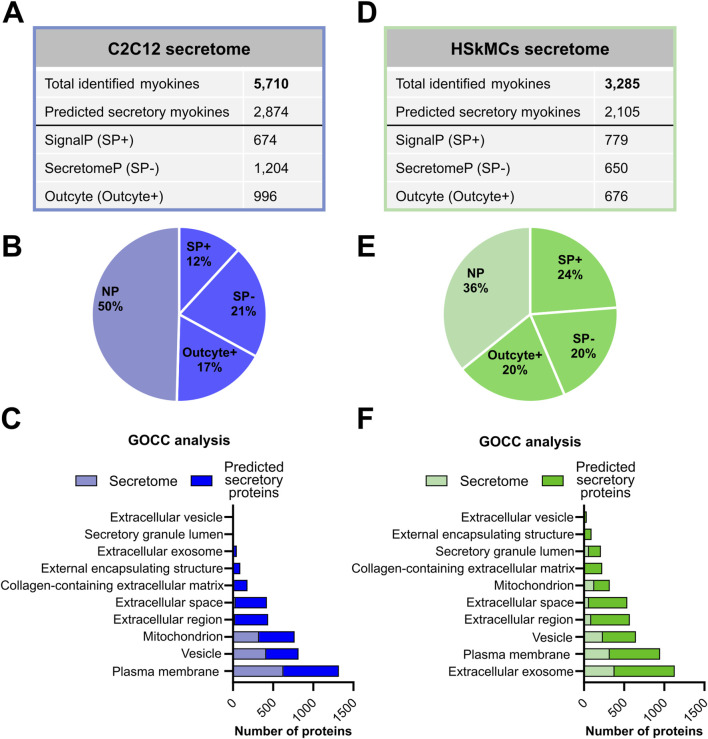
Bioinformatic prediction analyses of murine and human skeletal muscle secretomes. **(A)** C2C12 secretome results were analyzed using PD and subsequently, a subset of the secretome (“predicted secretory myokines”) was classified into classically and non-classically secreted proteins using SignalP 6.0, SecretomeP 2.0 and Outcyte 1.0. **(B)** Percentage of “predicted secretory myokines” from the murine C2C12 secretome. **(C)** GOCC analysis of murine C2C12 secretome and “predicted secretory myokines”. **(D)** The secretome from HSkMCs was categorized into classical and non-classical secretion types. **(E)** Percentage of “predicted secretory myokines” from HSkMCs secretome. **(F)** GOCC analysis of the HSkMCs secretome and “predicted secretory myokines”. Samples from C2C12 cells (*n* = 3) are demonstrated in blue and samples from HSkMCs from three subjects (*n* = 3) are displayed in green. CON: control, EPS: electrical pulse stimulation, GOCC: Gene Ontology cellular component, NP: non-predicted, SP+: signal peptide positive, SP−: signal peptide negative.

We aimed to compare the EPS-induced muscle secretomes of murine C2C12 cells and primary HSkMCs in order to shed light on the similarities and differences of the most frequently exercise-studied cell models. Therefore, C2C12 and human myotubes were subjected to acute low-frequency EPS for 6 hours, while control cells remained unstimulated. Proteomic profiling of the C2C12 muscle secretome resulted in the identification of 5,710 potential myokines, of which 2,874 proteins were predicted as secretory myokines using bioinformatic tools described in the methods section (Figure 3A). Moreover, 674 proteins (12%, SP+) were considered as classically secreted proteins, while 1,204 (21%, SP-) and another 996 myokines (17%, Outcyte+) were recognized as unconventionally secreted proteins (Figures 3A, B). Gene Ontology cellular component (GOCC) analysis revealed that most murine-secreted proteins were annotated as targeted to “plasma membrane proteins”, “vesicles” and “mitochondrial proteins” (Figure 3C). Furthermore, secretome analysis of HSkMCs identified 3,285 potential myokines in the CM of HSkMCs, of which 2,105 proteins were predicted to be secretory myokines (Figure 3D). Moreover, 779 proteins (24%, SP+) were considered as classically secreted proteins, while 650 (20%, SP-) and another 676 myokines (20%, Outcyte+) were recognized as unconventionally secreted proteins (Figures 3D, E). Interestingly, GOCC analysis revealed that most proteins in the human secretome were annotated as “extracellular exosome”, “plasma membrane” as well as “extracellular space” and “extracellular region” (Figure 3F).

### A direct comparison: secretome versus proteome analysis

**FIGURE 4:**
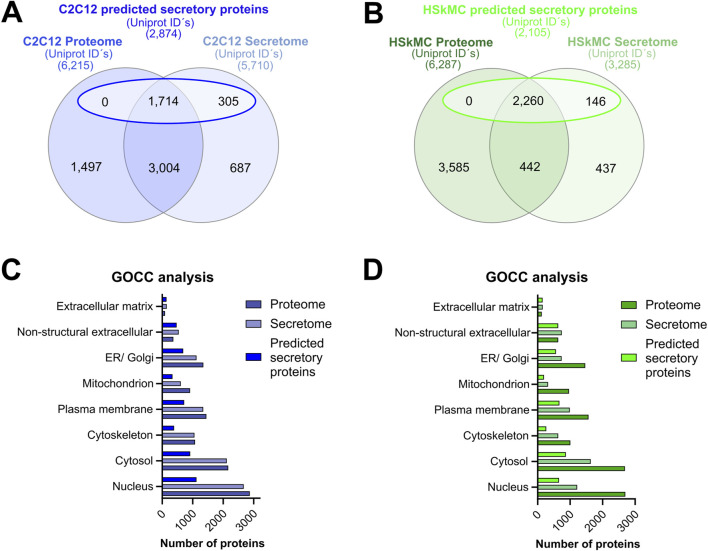
Secretome versus cellular proteome analyses. Alignment of proteome data with either total secretome or a subset of secretome data (“predicted secretory myokines”) from C2C12 cells **(A)** or HSkMCs **(B)**. GOCC analyses of C2C12 **(C)** and HSkMCs **(D)** proteome, secretome and “predicted secretory myokines” data was conducted. Venn analyses were performed based on UniprotKB IDs for “*mus musculus*” and “*homo sapiens*”. Data shown in blue represent C2C12 cells (*n* = 3) while data displayed in green represent HSkMCs from three subjects (*n* = 3). GOCC: Gene Ontology cellular component.

Murine and human myotubes were harvested, lyzed and subjected to MS-based analysis as described in the methods section. Direct comparison of the murine and human muscle secretome and cellular proteome was performed as previously described in methods. The interspecies comparative analysis of the muscle secretomes revealed an overlap of 2,556 proteins that were present in the CM of both C2C12 cells and HSkMCs, while a greater number of proteins was secreted from C2C12 myotubes than from human myotubes (Supplemental Figure S1A). The murine and human cellular proteomes were quite similar as they shared 4,929 common proteins (Supplemental Figure S1B). Furthermore, comparative analysis of the muscle secretome (5,710 proteins) and proteome (6,215 proteins) of C2C12 myotubes was performed, which resulted in an overlap of 4,718 proteins, showing that 83% of all secreted proteins were also detected in the cellular proteome (Figure 4A). Also, a large number of proteins (1,714) previously categorized as “predicted secretory myokines” (a subset of the secretome) overlapped with the C2C12 proteome (Figure 4A). Similar results were observed for the HSkMCs. The secretome (3,285) and the proteome (6,287) of human myotubes resulted in an overlap of 2,702 proteins, which corresponds to 82% of all secreted proteins that were also detected in the cellular proteome (Figure 4B). The “predicted secretory myokines” of the human muscle secretome overlapped with the human proteome with 2,260 proteins (Figure 4B). To gain insight into the protein distribution within versatile GO terms for intracellular and extracellular components, GOCC analysis was performed for both cell models. Interestingly, the ratios of proteins annotated for intracellular GO terms such as “nucleus, cytosol and cytoskeleton” were nearly identical in the murine secretome and proteome (Figure 4C), while the number of proteins for these GO terms in the human secretome was remarkably lower in comparison to the human proteome (Figure 4D). The number of proteins annotated as GO “extracellular matrix” and “non-structural extracellular” proteins was comparable in the secretome and proteome dataset of both C2C12 cells and HSkMCs (Figures 4C, D). To determine the abundance distribution of the proteins detected in the secretome and proteome and whether the same proteins are similarly distributed in C2C12 cells and HSkMCs, the top 100 candidates were ranked according to their PSM. We observed that 52% of the top 100 abundant proteins were present in both murine secretome and proteome. Likewise, the human secretome and proteome shared 47% of the top 100 proteins (Supplemental Table S2; Supplemental Figure S2).

### EPS-induced proteins in the murine and human muscle secretome

**FIGURE 5:**
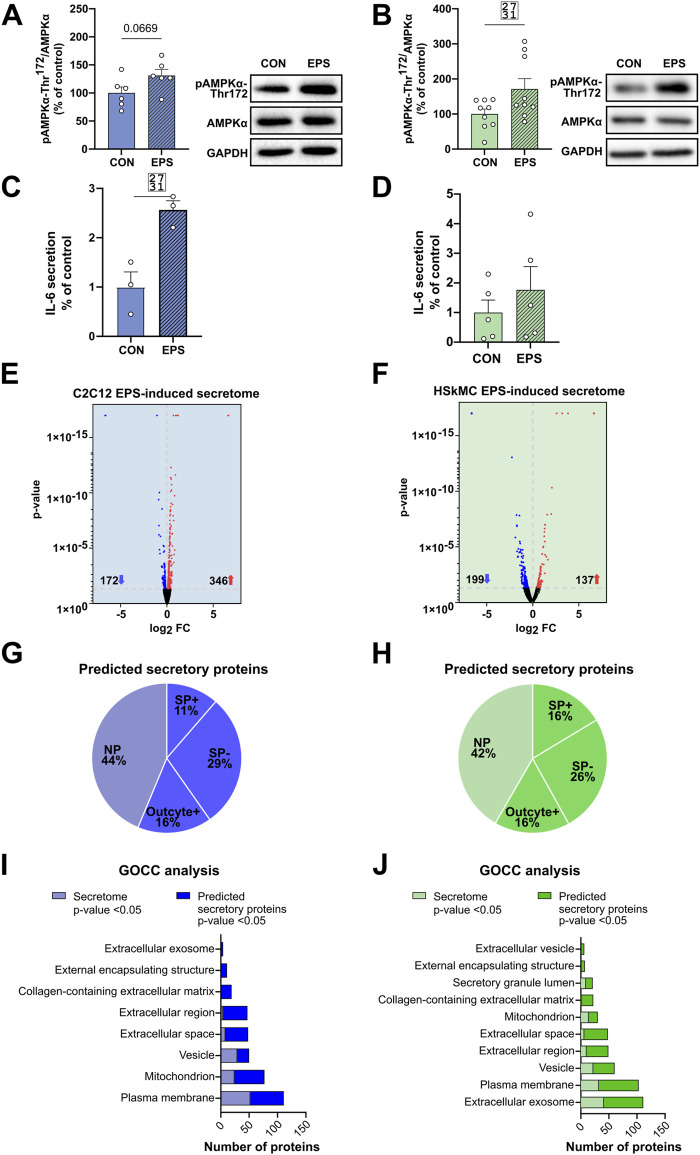
EPS-induced differentially regulated proteins in murine und human muscle secretome. Cell lysates from C2C12 myotubes **(A)** or from human myotubes from three individuals **(B)** were analyzed for changes in phosphorylation of AMPKα following EPS by immunoblotting with AMPKα-Thr^172^ specific antibody. IL-6 protein secreted in the CM of C2C12 **(C)** and human myotubes **(D)** was measured using multiplex immunoassay. Volcano plot analysis of 172 downregulated and 346 upregulated myokines in the C2C12 secretome **(E)** Volcano plot analysis of 199 downregulated and 137 upregulated myokines in the HSkMCs secretome **(F)**. Bioinformatic prediction analysis of differentially significantly regulated proteins from C2C12 secretome **(G)** or human secretome **(H)**. Gene Ontology cellular component (GOCC) analysis of differentially significantly regulated proteins classified as SP+, SP−, Outcyte+ from C2C12 secretome **(I)** or human secretome **(J)**. Phosphorylation signals in immunoblot analysis were normalized to GAPDH protein abundance and total AMPKα. Data are means ± SEM from C2C12 cells (*n* = 6) **(A)** and HSkMC **(B)** from three individuals (*n* = 3) and were analyzed by unpaired t-test (Welch´s correction), *p < 0.05 vs. CON. Data from C2C12 cells (*n* = 3) are shown in blue and data of HSkMCs from three subjects (*n* = 3) are presented in green. Striped bars represent EPS-stimulated cells. EPS: Electrical pulse stimulation, GOCC: Gene Ontology cellular component, NP: non-predicted, SP+: signal peptide positive, SP-: signal peptide negative, FC: fold change, SEM: standard error of the mean, IL-6: interleukin-6.

The intracellular energy sensor AMPK is activated in response to muscle contraction, therefore immunoblotting of AMPK activity at its main regulatory phosphorylation site (Thr-172) was performed to validate our acute low-frequency EPS protocol. Phosphorylation of AMPKα at Thr-172 was significantly increased after 6 hours of EPS in HSkMCs. Moreover, an increase was also observed in C2C12 cells but without reaching statistical significance (Figures 5A, B). The presence of known secreted myokines such as IL-6 in both murine and human muscle secretomes after EPS was confirmed by multiplex immunoassay (Figures 5C, D). Furthermore, MS datasets were analyzed as described in methods and filtered for significantly regulated myokines (*p*-value < 0.05). EPS induced changes in 518 myokines quantified in the CM of C2C12 myotubes, of which 172 proteins were significantly downregulated while 346 proteins were significantly upregulated (Figure 5E). In comparison, 336 myokines were differentially regulated by EPS in the CM of primary human myotubes, of which 199 proteins were significantly downregulated and 137 proteins were significantly upregulated (Figure 5F). Interestingly, comparative analysis of murine and human muscle secretomes revealed that EPS induced different myokine secretion profiles in both cell models, as indicated by a moderate overlap of myokines (40 proteins) in both secretomes (Supplemental Figure S3). Bioinformatic analysis revealed that 56% of the regulated myokines in the CM of C2C12 cells were considered as “predicted secretory myokines”, of which 11% were categorized as classically secreted proteins (SP+) and 45% as unconventionally secreted proteins (SP-, 29%; Outcyte+, 16%) (Figure 5G). Similar results were observed for HSkMCs. Bioinformatics predicted 58% of the myokines in the CM as secreted proteins, of which 16% were considered as classically secreted proteins (SP+) and 42% (SP-, 26%; Outcyte+, 16%) as unconventionally secreted proteins (Figure 5H). All EPS-induced classically secreted proteins in the murine secretome are listed in Table 1 and in the human secretome in Table 2, ranked according to their *p*-value (<0.05), while all unconventionally secreted proteins are listed in Supplemental Tables S3, S4. GOCC analysis of contraction-induced myokines in the CM of C2C12 myotubes proposed enrichment of proteins annotated for “plasma membrane”, “mitochondrion”, “vesicle”, “extracellular space” and “region” (Figure 5I). In contrast, contraction-induced myokines in the CM of HSkMCs were enriched for the GO terms “extracellular exosomes”, “plasma membrane”, “vesicle”, “extracellular region” and “extracellular space” (Figure 5J).

**TABLE 1:** EPS-induced differentially regulated and classically secreted proteins in CM from murine muscle secretome.

UniProtKB	Gene symbol	Protein description	p-value	Log2FC	Secretion
P58022	Loxl2	Lysyl oxidase homolog 2	1.00E-17	6.64	SP+
Q91WU0	Ces1f	Carboxylesterase 1F	1.00E-17	6.64	SP+
P10923	Spp1	Osteopontin	1.00E-17	1.21	SP+
O09161	Casq2	Calsequestrin-2	1.00E-17	0.96	SP+
Q9Z0M6	Adgre5	Adhesion G protein-coupled receptor E5	1.00E-17	1.13	SP+
Q8R180	Ero1a	ERO1-like protein alpha	1.00E-17	−6.64	SP+
Q9CQU0	Txndc12	Thioredoxin domain-containing protein 12	1.00E-17	−6.64	SP+
Q61475	Cd55	Complement decay-accelerating factor, GPI-anchored	1.00E-17	−6.64	SP+
O09165	Casq1	Calsequestrin-1	1.82E-11	0.51	SP+
P19788	Mgp	Matrix Gla protein	7.62E-11	0.4	SP+
Q8CA71	Shisa4	Protein shisa-4	1.15E-08	0.67	SP+
P70158	Smpdl3a	Acid sphingomyelinase-like phosphodiesterase 3a	3.16E-08	0.75	SP+
P47931	Fst	Follistatin	9.65E-07	0.3	SP+
P50608	Fmod	Fibromodulin	3.13E-05	−0.75	SP+
Q06335	Aplp2	Amyloid beta precursor like protein 2	3.28E-04	0.25	SP+
P47880	Igfbp6	Insulin-like growth factor-binding protein 6	6.11E-04	0.21	SP+
Q8BNJ2	Adamts4	A disintegrin and metalloproteinase with thrombospondin motifs 4	6.65E-04	0.26	SP+
O54819	Tfpi	Tissue factor pathway inhibitor	9.56E-04	0.22	SP+
Q8K007	Sulf1	Extracellular sulfatase Sulf-1	1.38E-03	−0.28	SP+
P11688	Itga5	Integrin alpha-5	1.60E-03	−0.19	SP+
A6X935	Itih4	Inter alpha-trypsin inhibitor. heavy chain 4	1.68E-03	0.56	SP+
P29788	Vtn	Vitronectin	1.91E-03	0.22	SP+
O70326	Grem1	Gremlin-1	1.93E-03	0.19	SP+
Q61982	Notch3	Neurogenic locus notch homolog protein 3	2.80E-03	0.2	SP+
Q9QZJ6	Mfap5	Microfibrillar-associated protein 5	3.47E-03	0.19	SP+
Q8K297	Colgalt1	Procollagen galactosyltransferase 1	4.98E-03	−0.35	SP+
P21237	Bdnf	Brain-derived neurotrophic factor	6.40E-03	−0.32	SP+
P01887	B2m	Beta-2-microglobulin	6.77E-03	0.15	SP+
Q00560	Il6st	Interleukin-6 receptor subunit beta	8.98E-03	0.52	SP+
P15379	Cd44	CD44 antigen	1.03E-02	−0.17	SP+
P12850	Cxcl1	Growth-regulated alpha protein	1.08E-02	0.19	SP+
Q80T3	Adgrl3	Adhesion G protein-coupled receptor L3	1.10E-02	0.17	SP+
Q9CYK2	Qpct	Glutaminyl-peptide cyclotransferase	1.16E-02	0.19	SP+
O08746	Matn2	Matrilin-2	1.22E-02	−0.16	SP+
O08807	Prdx4	Peroxiredoxin-4	1.28E-02	−0.24	SP+
O35664-3	Ifnar2	Isoform 3 of Interferon alpha/beta receptor 2	1.57E-02	0.17	SP+
O35887	Calu	Calumenin	2.01E-02	−0.16	SP+
P11087	Col1a1	Collagen alpha-1(I) chain	2.34E-02	−0.15	SP+
Q05186	Rcn1	Reticulocalbin-1	2.45E-02	−0.15	SP+
Q61810	Ltbp3	Latent-transforming growth factor beta-binding protein 3	2.57E-02	0.14	SP+
Q9ER41	Tor1b	Torsin-1B	2.65E-02	−0.18	SP+
Q60994	Adipoq	Adiponectin	2.68E-02	0.12	SP+
P21460	Cst3	Cystatin-C	2.85E-02	0.12	SP+
P15208	Insr	Insulin receptor	2.90E-02	0.39	SP+
Q62059-2	Vcan	Isoform V1 of Versican core protein	2.94E-02	−0.17	SP+
Q9JM99	Prg4	Proteoglycan 4	3.24E-02	0.25	SP+
A2AVA0	Svep1	Sushi, von Willebrand factor type A, EGF and pentraxin domain-containing protein 1	3.51E-02	−0.18	SP+
Q501P1	Fbln7	Fibulin-7	3.52E-02	0.28	SP+
P04925	Prnp	Major prion protein	3.54E-02	0.13	SP+
Q9Z1W4	Gdf11	Growth/differentiation factor 11	3.58E-02	0.38	SP+
P16882-2	Ghr	Isoform 2 of Growth hormone receptor	3.77E-02	0.19	SP+
Q8VHI3	Pofut2	GDP-fucose protein O-fucosyltransferase 2	3.95E-02	−0.16	SP+
P47879	Igfbp4	Insulin-like growth factor-binding protein 4	4.02E-02	0.12	SP+
P04756	Chrna1	Acetylcholine receptor subunit alpha	4.09E-02	−0.32	SP+
P19324	Serpinh1	Serpin H1	4.10E-02	−0.14	SP+
Q9Z121	Ccl8	C-C motif chemokine 8	4.16E-02	0.15	SP+
Q8CG19	Ltbp1	Latent-transforming growth factor beta-binding protein 1	4.19E-02	0.13	SP+
P28798	Grn	Progranulin	4.27E-02	0.11	SP+
Q9Z0J0	Npc2	NPC intracellular cholesterol transporter 2	4.41E-02	0.11	SP+

**TABLE 2:** EPS-induced differentially regulated and classically secreted proteins in CM from human muscle secretome.

UniProtKB	Gene symbol	Protein description	*p*-value	Log2FC	Secretion
P15309	ACP3	Prostatic acid phosphatase	1.00E-17	6.64	SP+
P23280	CA6	Carbonic anhydrase 6	1.00E-17	3.83	SP+
P02458	COL2A1	Collagen alpha-1(II) chain	1.00E-17	−6.64	SP+
Q02487-2	DSC2	Isoform 2B of Desmocollin-2	1.00E-17	6.64	SP+
Q16610-2	ECM1	Isoform 2 of Extracellular matrix protein 1	1.00E-17	6.64	SP+
A8MVW0	FAM171A2	Protein FAM171A2	1.00E-17	6.64	SP+
P25445	FAS	Tumor necrosis factor receptor superfamily member 6	1.00E-17	−6.64	SP+
O95867	LY6G6C	Lymphocyte antigen 6 complex locus protein G6c	1.00E-17	6.64	SP+
Q9Y639	NPTN	Neuroplastin	1.00E-17	−6.64	SP+
Q15063-7	POSTN	Isoform 7 of Periostin	1.00E-17	−6.64	SP+
P07602-3	PSAP	Isoform Sap-mu-9 of Prosaposin	1.00E-17	−6.64	SP+
Q9HB40	SCPEP1	Retinoid-inducible serine carboxypeptidase	1.00E-17	6.64	SP+
P49908	SELENOP	Selenoprotein P	1.00E-17	6.64	SP+
P20062	TCN2	Transcobalamin-2	1.00E-17	−6.64	SP+
P24821-4	TNC	Isoform 4 of Tenascin	1.00E-17	6.64	SP+
P02766	TTR	Transthyretin	1.00E-17	−6.64	SP+
P13500	CCL2	C-C motif chemokine 2	1.25E-08	1.57	SP+
Q13361	MFAP5	Microfibrillar-associated protein 5	1.48E-05	−1.57	SP+
P13667	PDIA4	Protein disulfide-isomerase A4	1.86E-05	−1.1	SP+
P07093	SERPINE2	Glia-derived nexin/Protease Nexin I (PN-I)	2.15E-04	−1.23	SP+
O95969	SCGB1D2	Secretoglobin family 1D member 2	3.43E-04	1.02	SP+
Q86WD7	SERPINA9	Serpin A9	1.09E-03	0.83	SP+
Q9BXX0	EMILIN2	EMILIN-2	2.31E-03	−1.15	SP+
P08138	NGFR	Tumor necrosis factor receptor superfamily member 16	7.48E-03	0.67	SP+
P14625	HSP90B1	Endoplasmin	8.58E-03	−0.68	SP+
Q15517	CDSN	Corneodesmosin	9.32E-03	0.63	SP+
Q9H4F8	SMOC1	SPARC-related modular calcium-binding protein 1	9.59E-03	0.61	SP+
P08648	ITGA5	Integrin alpha-5	1.02E-02	−0.87	SP+
P08246	ELANE	Neutrophil elastase	1.03E-02	0.64	SP+
P12111	COL6A3	Collagen alpha-3(VI) chain	1.04E-02	−0.67	SP+
O75339	CILP	Cartilage intermediate layer protein 1	1.18E-02	1.05	SP+
Q6E0U4	DMKN	Dermokine OS = *Homo sapiens* OX = 9606 GN = DMKN PE = 1 SV = 3	1.25E-02	0.96	SP+
P0DUB6	AMY1A	Alpha-amylase 1A	1.25E-02	0.66	SP+
Q8TCT8	SPPL2A	Signal peptide peptidase-like 2A	1.26E-02	0.63	SP+
Q03405	PLAUR	Urokinase plasminogen activator surface receptor	1.29E-02	0.83	SP+
P18065	IGFBP2	Insulin-like growth factor-binding protein 2	1.38E-02	−0.64	SP+
Q14703	MBTPS1	Membrane-bound transcription factor site-1 protease	1.41E-02	0.66	SP+
O43854	EDIL3	EGF-like repeat and discoidin I-like domain-containing protein 3	1.51E-02	−0.76	SP+
P49862	KLK7	Kallikrein-7	1.54E-02	0.62	SP+
O60911	CTSV	Cathepsin L2	1.57E-02	0.61	SP+
Q4V9L6	TMEM119	Transmembrane protein 119	1.77E-02	−0.89	SP+
Q08554-2	DSC1	Isoform 1B of Desmocollin-1	2.07E-02	0.58	SP+
Q9H1E1	RNASE7	Ribonuclease 7	2.11E-02	0.57	SP+
Q96S86	HAPLN3	Hyaluronan and proteoglycan link protein 3	2.21E-02	0.65	SP+
P05067	APP	Amyloid-beta precursor protein	2.26E-02	−0.59	SP+
P43307	SSR1	Translocon-associated protein subunit alpha	2.28E-02	0.85	SP+
Q9Y2I2	NTNG1	Netrin-G1	2.60E-02	0.88	SP+
Q9P121-4	NTM	Isoform 4 of Neurotrimin	2.66E-02	0.55	SP+
P25311	AZGP1	Zinc-alpha-2-glycoprotein	2.71E-02	0.55	SP+
Q9UMX5	NENF	Neudesin	3.56E-02	0.67	SP+
Q8WWX9	SELENOM	Selenoprotein M	3.81E-02	0.52	SP+
Q6Y288	B3GLCT	Beta-1,3-glucosyltransferase	3.82E-02	0.83	SP+
O43240	KLK10	Kallikrein-10	3.99E-02	0.63	SP+
P12273	PIP	Prolactin-inducible protein	4.40E-02	0.5	SP+
Q07507	DPT	Dermatopontin	4.45E-02	0.51	SP+

### Alignment of murine and human secretome data with the literature

**FIGURE 6:**
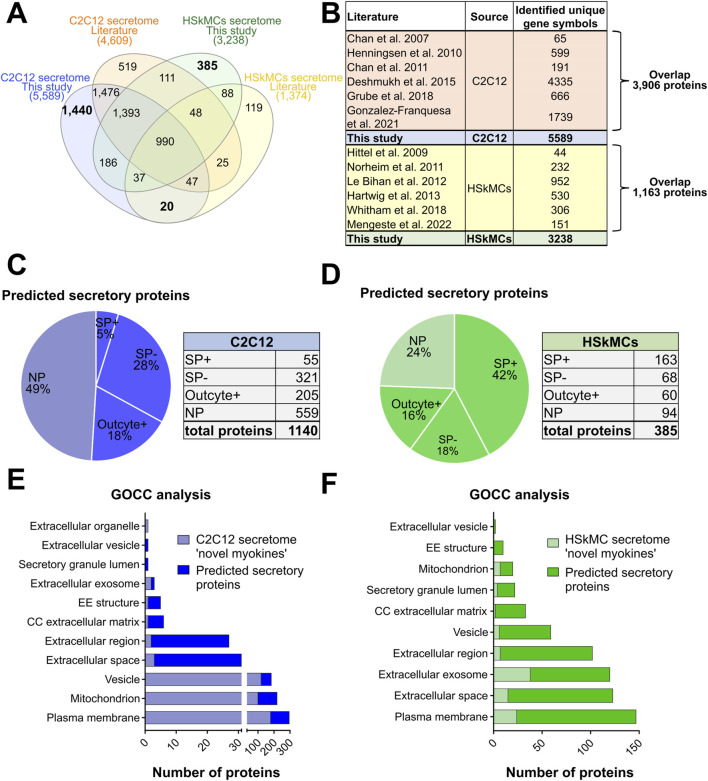
Comparison of this secretome study with previous MS-based secretome studies from the literature. **(A)** Venn diagram shows the alignment of secretome data of C2C12 cells and HSkMCs from this study compared to other MS-based secretome studies from the literature. **(B)** Table provides an overview of the literature included in the comparative analysis of C2C12 cells and HSkMCs and shows the overlap between our data and published data. Bioinformatic secretion type prediction analysis of newly described proteins in the C2C12 secretome **(C)** and HSkMCs secretome (D). GOCC analysis of the novel described proteins from the C2C12 secretome **(E)** and from the HSkMCs secretome **(F)**. Data from this study are displayed in blue for C2C12 cells (*n* = 3) and in green for HSkMCs from three subjects (*n* = 3). Data from the literature on C2C12 cells is represented in orange and from HSkMCs in yellow. GOCC: Gene Ontology cellular component, NP: non-predicted, SP+: signal peptide positive, SP−: signal peptide negative.

Differences in the composition of secretomes may result from using different cell types, culture conditions, sample preparation techniques and MS protocols. We therefore sought to compare our data with former secretome analyses and conducted a comprehensive analysis of the literature as previously described in methods. Our comparative analysis revealed that 1,440 myokines identified in the CM of C2C12 myotubes in this study have not yet been described in the literature. Furthermore, additional 20 proteins were identified in our C2C12 secretome that have been previously only identified in human secretome studies from the literature, while further 186 proteins were found exclusively in the murine and humane secretome of this study. Comparison with the literature on human cells resulted in the identification of 385 novel myokines in the CM of human myotubes that were unique to this study and had not been described in other MS-based studies. Moreover, 1,393 common proteins were found in the human and murine secretome of this study as well as in previously characterized murine secretome data from the literature. Additionally, 111 myokines were discovered in the human secretome in this study that were previously only described in the literature for C2C12 cells (Figure 6A). In this study, we confirmed the presence of known secreted myokines such as IL-6, decorin, desmin, SPARC and VEGFA in both murine and human muscle secretomes. Interestingly, other known myokines such as follistatin (FST), meteorin (METRN), sestrin-1 (SESN1) and insulin-like growth factor I (IGF-1) were only detected in the CM of C2C12 cells, whereas others such as leukemia inhibitory factor (LIF) were exclusively identified in the CM of HSkMCs (Supplemental Table S5). All MS-based secretome studies resulting from the literature research as described in “cross-species comparisons and literature searches” are shown in Figure 6B. The comparison of MS data from this study with data from the literature from C2C12 cells revealed an overlap of 3,906 myokines, while the comparison of data from HSkMCs revealed an overlap of 1,163 proteins (Figure 6B). Next, bioinformatic prediction analyses were performed to obtain information on the newly identified myokines, which were first described in this study (Figures 6C–F). Of the newly identified myokines in the murine muscle secretome, 51% were identified as “predicted secretory myokines'', of which a minority (5%) were secreted via the classical secretion pathway (SP+), while the majority (46%) were secreted via UPS pathways (SP- and Outcyte+) (Figure 6C). In contrast, 42% of the newly identified myokines of the human secretome were predicted to be “classically secreted proteins” (SP+), while 34% were secreted via UPS pathways (SP-, Outcyte+) (Figure 6D). GOCC analysis of the novel myokines revealed that the majority of proteins in both the murine and human secretome were annotated with the GO term “plasma membrane”. In the murine secretome, most proteins were annotated as “mitochondrion” and “vesicle”, while in the human secretome most common GO terms were “extracellular space”, “extracellular exosome” and “extracellular region” (Figures 6E, F).

## Discussion

Previous studies have analyzed EPS-induced myokine secretion from cultured muscle cells. However, several cell types and contraction protocols have been utilized, which may at least partly explain the different outcomes of the secretome studies (Nikolić et al., 2017). In our study, we have compared the myokine secretion profiles of the two most commonly used skeletal muscle models, murine C2C12 cells and primary human myotubes from different donors, in response to acute (6 h) low-frequency (1 Hz) EPS stimulation, which is thought to mimic a single bout of exercise and allows investigating the acute response of the cells (Christensen et al., 2015). Subsequently, we performed a non-targeted proteomics approach using high-resolution MS to determine the secretome profiles.

Collectively, our secretome analysis revealed an unprecedented number of potentially secreted proteins in the conditioned media of skeletal muscle cells. Our analysis revealed that C2C12 myotubes secreted a substantially higher number of proteins (5,710) compared to primary human myotubes (3,285). Both secretomes showed an overlap in 2,556 proteins, with 80% of human myokines also detected in the murine secretome. Interestingly, GOCC analysis revealed that most myokines in the human secretome were targeted to “extracellular exosomes” (>1,000 proteins), whereas in the murine secretome the number of myokines for this annotation was notably lower (<50 proteins). These data demonstrate that, at least for short-term EPS, primary HSkMCs exhibit a more complex secretory response to EPS than cultured C2C12 cells regarding extracellular exosomes. As C2C12 cells and primary HSkMCs cells did not differ in morphology, gene expression profiles during myogenesis and cell integrity/viability after EPS, we propose that both cell types exhibit distinct differences in myokine secretion. In fact, we observed that twice as many myokines were secreted via the classical secretory pathway in HSkMCs (24%) compared to C2C12 cells (12%), whereas the percentage of unconventionally secreted myokines was similar in both species (38%–40%), emphasizing that C2C12 cells may utilize different secretion pathways than HSkMCs. However, bioinformatic tools such as SignalP, SecretomeP and Outcyte may have limitations to identify *bonafide* secreted proteins across different species, requiring validation through different experimental approaches, such as to Brefeldin A treatment that blocks protein secretion via the classical secretory pathway in both species (Misumi et al., 1986). Interestingly, a recent study using Brefeldin A treated C2C12 cells confirmed that SignalP detects classically secreted proteins with 93% certainty (Grube et al., 2018).

Our study identified 1,440 novel murine myokines and 385 novel human myokines secreted by myotubes, which have previously not been described in the literature. This increase in identified proteins is likely owed to an enhanced sensitivity of our MS analysis. In addition, different cell origins and culture conditions may also contribute to the observed differences in identified proteins. Indeed, the data used for the literature comparison were obtained from studies of murine (Henningsen et al., 2010; Chan et al., 2011) and human myoblasts (Le Bihan et al., 2012) during myogenesis, murine (Chan et al., 2007; 2011; Grube et al., 2018) and human myotubes (Hartwig et al., 2014), murine myotubes exposed to palmitate (Deshmukh et al., 2015) or human myotubes generated from obese subjects (Hittel et al., 2009) as well as murine (Gonzalez-Franquesa et al., 2021) or human myotubes (Mengeste et al., 2022) exposed to chronic low-frequency EPS and myotubes of healthy donors who have undergone a pre- and post-exercise intervention (Norheim et al., 2011; Whitham et al., 2018).

Acute low-frequency EPS induced 518 differentially regulated myokines in the C2C12 secretome, while 336 myokines were differentially regulated in the human secretome. Importantly, our bioinformatic analysis revealed that most EPS-regulated proteins were secreted via the UPS pathways (42%–45%) in both species. Hence, EPS-induced changes in the murine and human muscle secretome were similar concerning the secretory pathways, however, the overlap of EPS-regulated myokines in both species was rather moderate (40 common myokines). Previous studies using non-targeted MS found a considerably lower number, approx. 75–150 of EPS-regulated myokines in murine (Gonzalez-Franquesa et al., 2021) and human secretomes (Mengeste et al., 2022). Both studies used similar low-frequency EPS protocols, but for a duration of 24 h instead of 6 h in the present study, thus resembling rather chronic exposure to muscle contraction instead of an acute bout of exercise. Chronic EPS stimulation may also affect secretomes through adaptive mechanisms and feedback loops related to myokine secretion, metabolite availability, cell integrity and other factors, adding to the complexity of comparing differences in secretome profiles.

In addition to well established myokines such as IL-6, we identified numerous other proteins secreted by myotubes upon EPS/contraction such as GREM1, OPN1, CTRP3 and PSAP which may play important roles in metabolism and organ cross talk. GREM1 (Gremlin-1), a BMP antagonist initially reported to be secreted from adipose cells, has been implicated in inhibiting insulin signaling and found to be increased in type 2 diabetes (Hedjazifar et al., 2020). While visceral fat derived GREM1 has been suggested to regulate hepatocellular senescence in NAFLD (Baboota et al., 2022), its role as a myokine is yet unknown. Similarly, OPN1 (Osteopontin), an extracellular matrix protein and proinflammatory cytokine released from macrophages was found to modulate insulin sensitivity (Nomiyama et al., 2007). CTRP3 (C1q/TNF-related protein 3) is recognized as an adipokine involved in metabolic regulation, inflammation, and tissue repair and has been shown to improve hepatic glucose metabolism and insulin sensitivity (Guo et al., 2020). Notably, GREM1, OPN1 and CTRP3 were associated with cardioprotective effects following myocardial infarction or with attenuating cardiac dysfunction such as diabetic cardiomyopathy (Ma et al., 2017; Zhang et al., 2019; Kuprytė et al., 2024). Lastly, PSAP (prosaposin) was recently described as a novel secretory factor stimulating thermogenic gene expression and inducing mitochondrial respiration in adipose cells (Mittenbühler et al., 2023). Our discovery that these molecules are secreted from myotubes in response to EPS/contraction implies that the health-enhancing benefits of exercise may be mediated by a significant number of different myokines. In summary, in our study we have comprehensively analyzed myokine secretion of sedentary and contracted murine and human skeletal muscle cell models. While both cell models are relatively similar, we found cell type-specific differences in cellular compartments involved in myokine secretion. These differences, as well as the differences in myokine signatures, could relate to evolutionary divergence or cell type-specific differences in regulatory mechanisms. Our analysis has identified numerous potential novel myokines that may contribute to the health-promoting effects of exercise in humans and may serve as guidance and knowledge resource for future studies in exercise physiology.

## Data Availability

The mass spectrometry proteomics data presented in the study are deposited to the ProteomeXchange Consortium via the PRIDE with the dataset identifier PXD058612 and PXD059003.

## Supplementary material

SUPPLEMENTARY IMAGE 1 Venn diagram analyses showing interspecies comparison of muscle secretomes and cellular proteomes. **(A)** Comparison of murine and human secretomes was conducted based on gene symbols. **(B)** Comparison of murine and human proteomes was performed based on gene symbols. Venn analyses from C2C12 cells (n = 3) are shown in blue and from HSkMCs from three different donors (n = 3) in green

SUPPLEMENTARY IMAGE 2 The top 100 most abundant proteins from cellular proteome and secretome data, ranked by their PSM. **(A)** Venn diagram analysis shows the overlap of the common top 100 proteins in the proteome and secretome analysis as well as for the ‘predicted secretory proteins’ of C2C12 cells. **(B)** Venn diagram analysis demonstrates the overlap of the common top 100 proteins’ in the proteome and secretome analysis as well as for the ‘predicted secretory proteins’ of HSkMCs. Venn analyses were performed based on UniprotKB accession number for mus musculus and homo sapiens. Data displayed in blue represent C2C12 cells (n = 3) while green data represents HSkMCs from three subjects (n=3). HSkMCs: human skeletal muscle cells, PSM: peptide spectrum match.

SUPPLEMENTARY IMAGE 3 Comparison of EPS-induced proteins in CM of C2C12 cells and primary HSkMCs. The EPS-induced secretome of C2C12 cells (n = 3) were compared with EPS-induced secretome of HSkMCs (n = 3). Skeletal muscle cells were exposed to six hours of EPS and subsequently the conditioned media was analyzed by MS. Venn diagram shows data from C2C12 cells (n = 3) in blue and of HSkMCs from three subjects (n = 3) in green. EPS: Electrical pulse stimulation, HSkMCs: human skeletal muscle cells, MS: mass spectrometry.

SUPPLEMENTARY IMAGE 4 Comparison of differentially regulated proteins in CM of C2C12 cells and primary HSkMCs induced by different EPS protocols. **(A)** Murine EPS-induced secretome data of C2C12 cells (n = 3) that were stimulated for six hours from this study compared to murine EPS-induced secretome data of C2C12 cells that were stimulated for 24 hours published by Gonzalez-Franquesa et al. 2021. **(B)** Human EPS-induced secretome data of HSkMCs that were stimulated for six hours from this study compared to murine EPS-induced secretome data of HSkMCs that were stimulated for 24 hours published by Mengeste et al. 2022. Venn diagram shows data from C2C12 cells (n = 3) in blue (light blue = this study, dark blue = literature) and of HSkMCs from three subjects (n = 3) in green (light green = this study, dark green = literature). EPS: Electrical pulse stimulation, HSkMCs: human skeletal muscle cells.

SUPPLEMENTARY TABLE 1 Primers used for RT-qPCR analysis for HSkMCs and C2C12 cells.

SUPPLEMENTARY TABLE 2 Top 100 candidates in the murine and human secretome and proteome ranked according to their peptide spectrum match (PSM).

SUPPLEMENTARY TABLE 3 EPS-induced differentially regulated unconventionally secreted proteins in the muscle secretome of C2C12 cells.

SUPPLEMENTARY TABLE 4 Predicted secretory proteins which are differentially regulated in the human muscle secretome following 6 h of EPS.

SUPPLEMENTARY TABLE 5 Comparison of our secretome study with previous MS-based studies from the literature.
