# Supplementary figures and images for: High-resolution analyses of the secretomes from murine C2C12 cells and primary human skeletal muscle cells reveal distinct differences in contraction-regulated myokine secretion
Source: Front Physiol. 2025 Mar 25;16:1549316. doi: 10.3389/fphys.2025.1549316 (PMC11975866; doi:10.3389/fphys.2025.1549316)

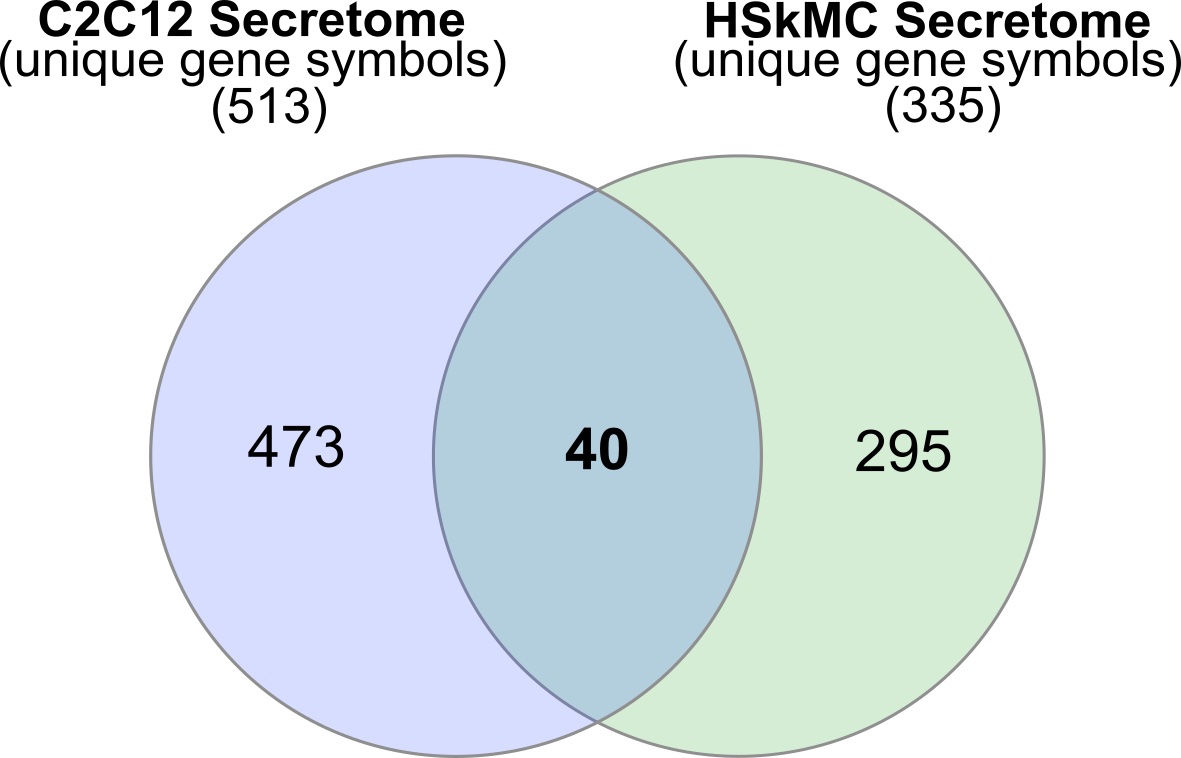

Supplement: Supplementary file 2 [file Image3.jpeg]

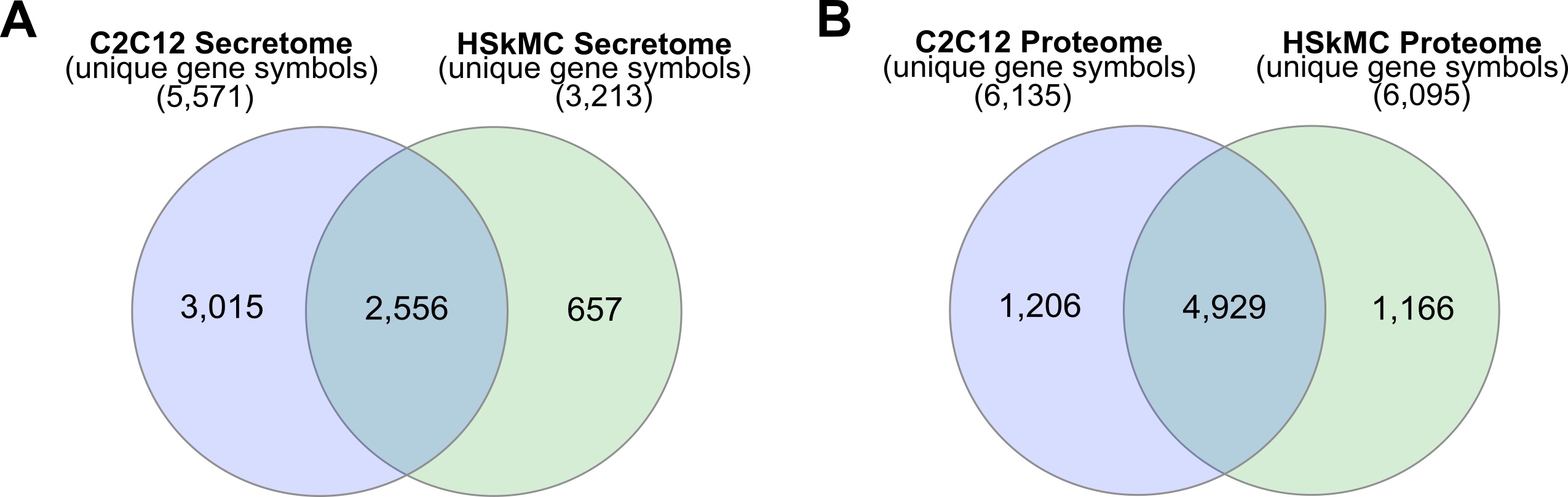

Supplement: Supplementary file 4 [file Image1.jpeg]

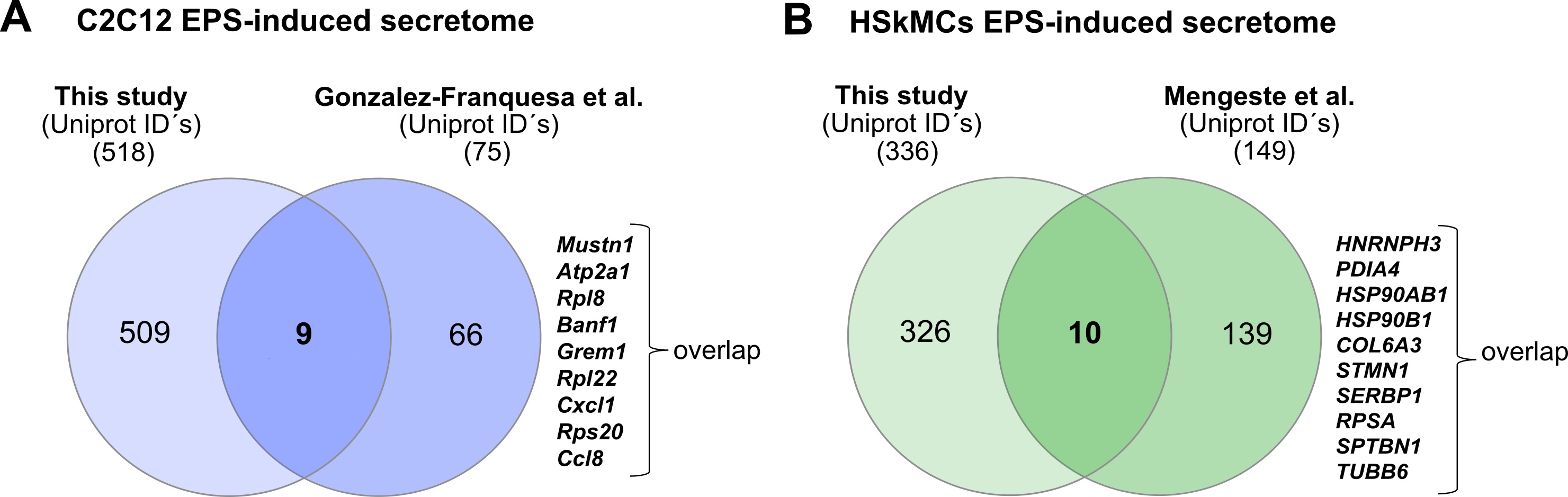

Supplement: Supplementary file 5 [file Image4.jpeg]

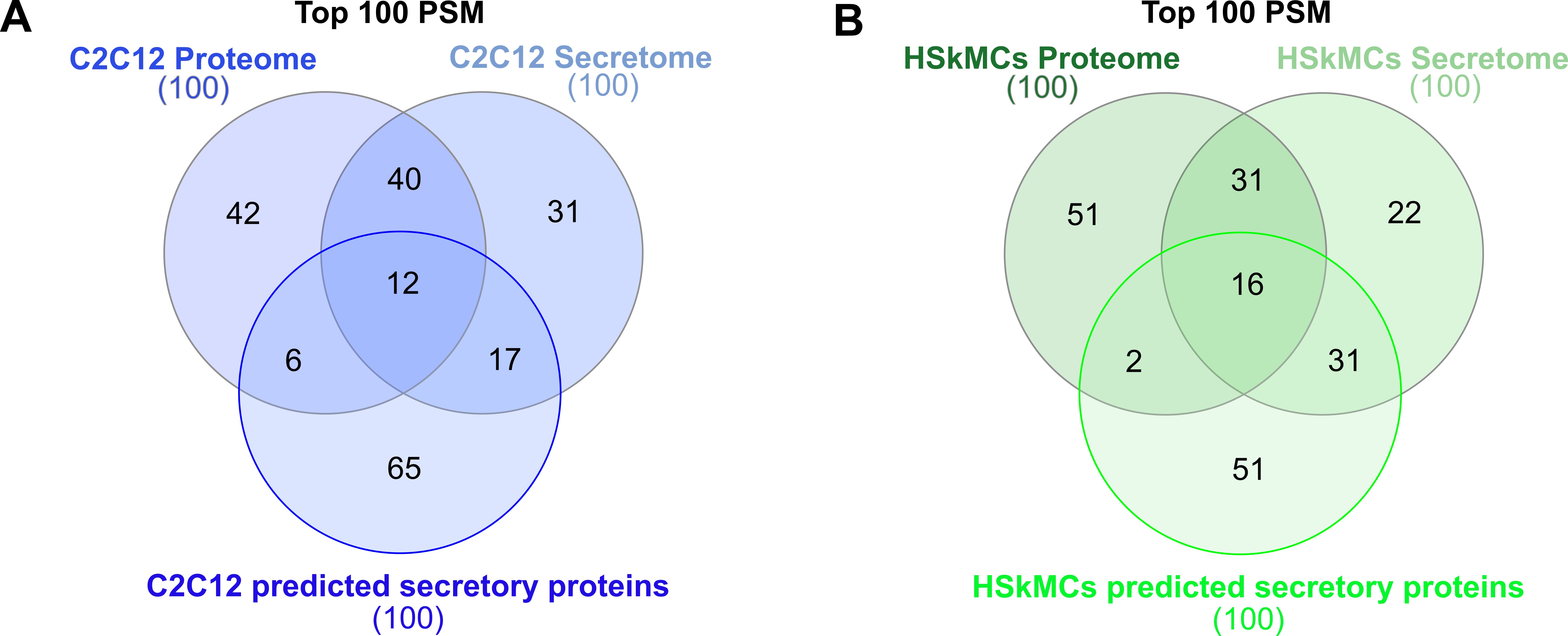

Supplement: Supplementary file 6 [file Image2.jpeg]
